# Multiple Neuritis from Arsenical Poisoning

**Published:** 1899-04

**Authors:** Isham Harrison

**Affiliations:** Deerbrook, Miss.


### MULTIPLE NEURITIS FROM ARSENICAL POISONING.

By ISHAM HARRISON, M.D.,

Deerbrook, Miss.

During the past year (1898) a number of cases of arsenical poisoning came under my observation which presented symptoms rarely mentioned in any text-book. They were all located on Mr. C.'s plantation, three miles from this place. Thirteen were negroes, three of whom speedily succumbed, and two whites, both of whom recovered. One after another was stricken down, until there remained only a few sound people on the place.

For some time the source of the poison was involved in mystery; but finally it was observed that whenever the patients ate meat their sufferings immediately became worse. Upon investigation the poison was found to be Paris green (arseniate copper), a can of which had occupied a place in Mr. C.'s cellar for ten years, in company with the boxes of meat. It had been used to exterminate cotton worms, which were a great pest at that time, but their visitations having ceased, the Paris green was relegated to a nook in the cellar and forgotten. The bottom of the can had rusted out, and the contents were scattered over the dirt floor, a quantity of the poison adhering to the sides of the nearest meat box.

Every person who ate this meat showed evidence of arsenical poisoning. Intense nausea, vomiting, and abdominal pain were usually the first symptoms, with puffy eyelids, and, in some instances, general edema. I do not think there was any diarrhea or dysentery, though I did not see all of the unfortunates. Some, at this point, abandoned their meat diet and escaped more serious trouble.

Three cases, all negroes and heavy meat-eaters, were afflicted with multiple neuritis. They suffered, constantly, from excruciating pains in their extremities, worse at night, and eventually complete extensor paralysis developed (wrist drop and foot drop), with total abolition of reflexes. Muscular atrophy soon followed, and the patients were confined to their beds and helpless for months. The soles of their feet were extremely tender and painful, having a mottled appearance and a soggy, rotten-Irish-potato feel to the sense of touch.

I wish to call attention to the fact, which was a feature of considerable interest to me, that in nearly every instance these people complained of tender soles and some could not walk—claiming to be perfectly well, and able to work, with this exception.

Pigmented spots were noticed on the bodies of some of the worst cases. The pulse rate ranged from 90 to 100, but there was never any elevation of temperature.

One of the patients, who was affected with peripheral neuritis, moved away and I lost sight of him; the other two, under the use of iodide potassium, strychnine, massage and electricity, made slow recoveries.

---

### CHANCROID IN ANIMALS.

Sapuppo.—Chancroid in animals (*Giorn. ital. d. mal. ven.*, v. 33, p. 43). Numerous inoculations of chancroids were made in rabbits, guinea-pigs and pigeons. Inflammatory lesions were produced but the Ducrey bacillus disappeared and only the ordinary pyogenic organisms were found. This new pus proved negative to man. The author believes the soft sore to be peculiar to man.—*The Dominion Medical Monthly.*

